# Treatment of a non-typical hepatic pseudolesion complicated by greatly elevated alpha fetoprotein: case report and literature review

**DOI:** 10.1186/1477-7819-11-238

**Published:** 2013-09-23

**Authors:** Xiao-Long Liu, Ling-Yan Zhang, Fu-Qiang Li, Yong-Hong Liang, Qing-Zhu Wei, Li-Xin Liu, Hai-Yan Cui

**Affiliations:** 1 Department of General Surgery, Third Affiliated Hospital of Southern Medical University, Guangzhou 510630, PR China; 2 Department of Radiology, Third Affiliated Hospital of Southern Medical University, Guangzhou 510630, PR China; 3 Department of General Surgery, First Affiliated Hospital, Zhejiang University School of Medicine, Hangzhou 310003, PR China; 4 Department of Pathology, Third Affiliated Hospital of Southern Medical University, Guangzhou 510630, PR China; 5 Department of Internal Medicine, Third Affiliated Hospital of Southern Medical University, Guangzhou 510630, PR China

**Keywords:** Hepatic pseudolesion, Alpha fetoprotein, Diagnosis

## Abstract

**Background:**

Hepatic pseudolesions detected by helical computed tomography (CT) are not rare, but it is difficult to make a final diagnosis when the hepatic lesion is complicated by the presence of greatly elevated alpha fetoprotein (AFP). Clinical treatment of non-typical hepatic pseudolesions complicated by greatly elevated AFP should confirm the diagnosis and minimize trauma.

**Case presentation:**

Non-invasive procedures including ultrasonography, CT, and micro-invasive digital subtraction angiography could not safely differentiate this lesion from a malignant focus when it was complicated by greatly elevated AFP. Laparoscopic hepatectomy was performed, and pathological analysis showed chronic hepatitis, nodular regenerative hyperplasia, focal nodular hyperplasia of the liver, and mild vascular malformation. The tissue was HbsAg(−), HbcAg(−), and AFP(+).

**Conclusion:**

Heightened awareness of hepatic pseudolesion complicated by primarily elevated AFP will help physicians avoid unnecessary invasive procedures. Hepatic biopsy is inevitable because of greatly elevated AFP. For suspected hepatic pseudolesion with elevated AFP, needle-core biopsy and follow-up surveillance instead of hepatectomy are recommended to find the source of AFP and make a final diagnosis of pseudolesion.

## Background

Hepatic pseudolesions adjacent to the falciform ligament have been observed using computed tomography (CT) [1], but no specific disease has been related to these pseudolesions [2,3]. When the hepatic lesion is complicated by elevated alpha fetoprotein (AFP), the image findings cannot be used to make a final clinical diagnosis [4]. It is unclear which procedures should be performed in this situation to confirm the diagnosis of hepatic pseudolesion.

Here we present a case of a non-typical hepatic pseudolesion observed on CT that was complicated by chronic hepatitis and elevated AFP. We aimed to identify rational clinical procedures that minimize trauma and that help diagnose a non-typical hepatic pseudolesion complicated by elevated AFP.

## Case presentation

**Figure 1:**
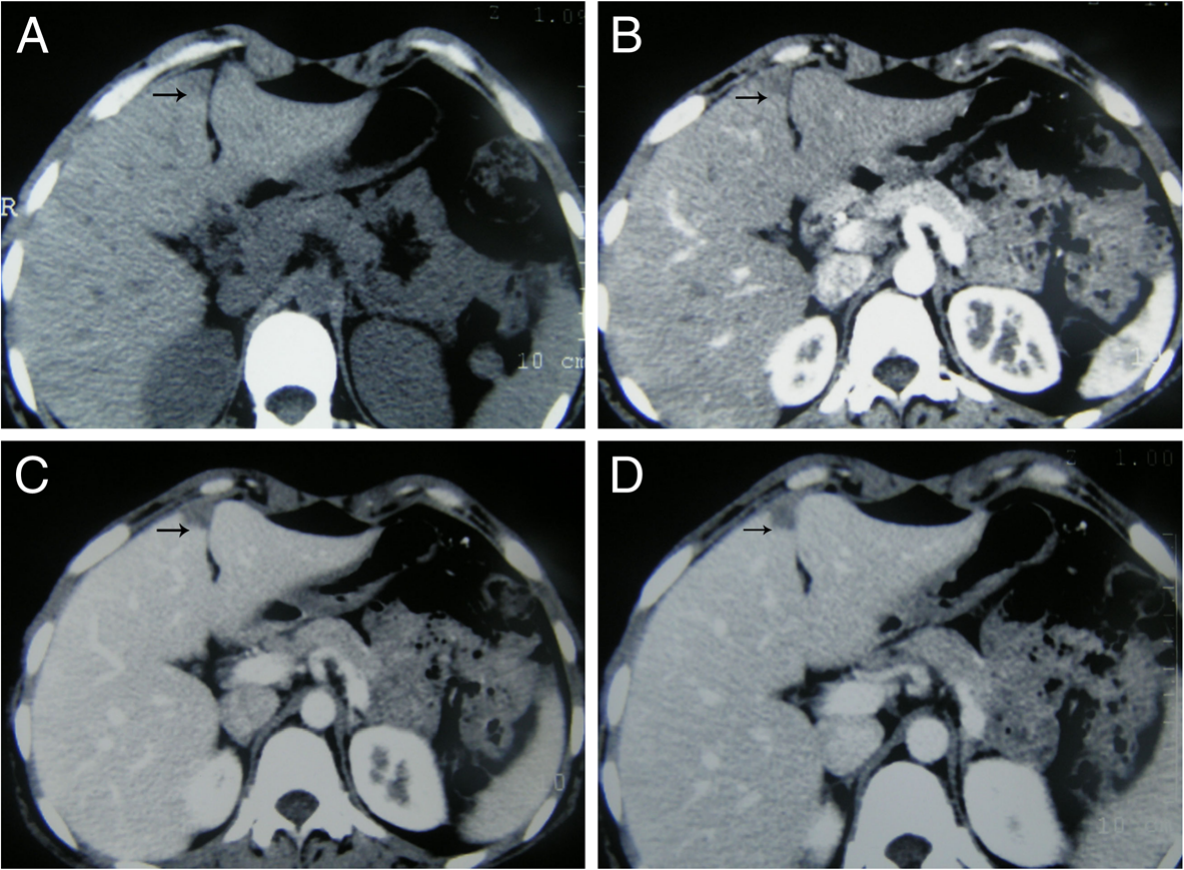
**Computed tomography (CT) findings.** In each panel, the arrow indicates the pseudolesion. **(A)** Unenhanced phase CT image. **(B)** Arterial-dominant phase CT image. **(C)** Portal-dominant phase CT image. **(D)** Equilibrium phase CT image.

A 23-year-old woman presented with elevated AFP during routine physical examination. There was no history of viral hepatitis, drug abuse, or abdominal surgery. Her AFP was 1,127 ng/ml, and she was negative for human chorionic gonadotropin, hepatitis B virus, hepatitis C virus, and hepatitis G virus. Her HbsAb was 748 IU/L. Her other biochemical measures were as follows: aspartate amino-transferase, 17 U/L; alanine aminotransferase, 13 U/L; albumin, 46 g/L; globulin, 31 g/L; and total bilirubin, 12.7 μmol/L. Diseases of the reproductive and urinary system that are associated with abnormal AFP levels were excluded. A lesion in the liver was not detected by ultrasonography, but we observed a blunt liver edge and rough echo patterns throughout the liver, suggesting chronic hepatic disease. Incremental helical CT was performed to characterize the hepatic disease. A suspected low-attenuated lesion of irregular shape was found in all phases of helical CT with a 32-row detector in section IV adjacent to the falciform ligament (Figure 1). A malignant diagnosis could not be ruled out by the CT images.

**Figure 2:**
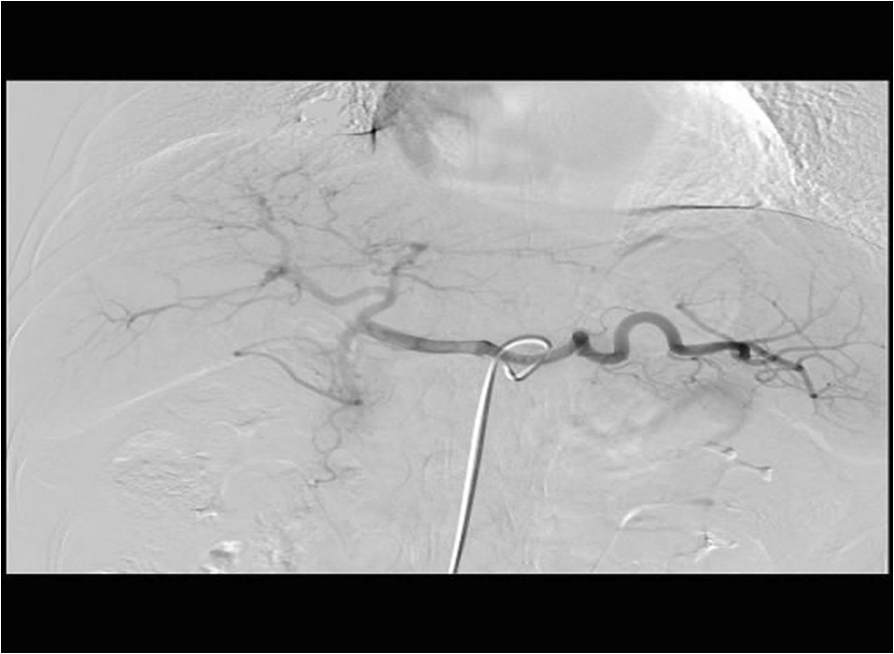
**Digital subtraction angiography using contrast enhancement.** The findings were not typical of a pseudolesion from vascular malformation.

**Figure 3:**
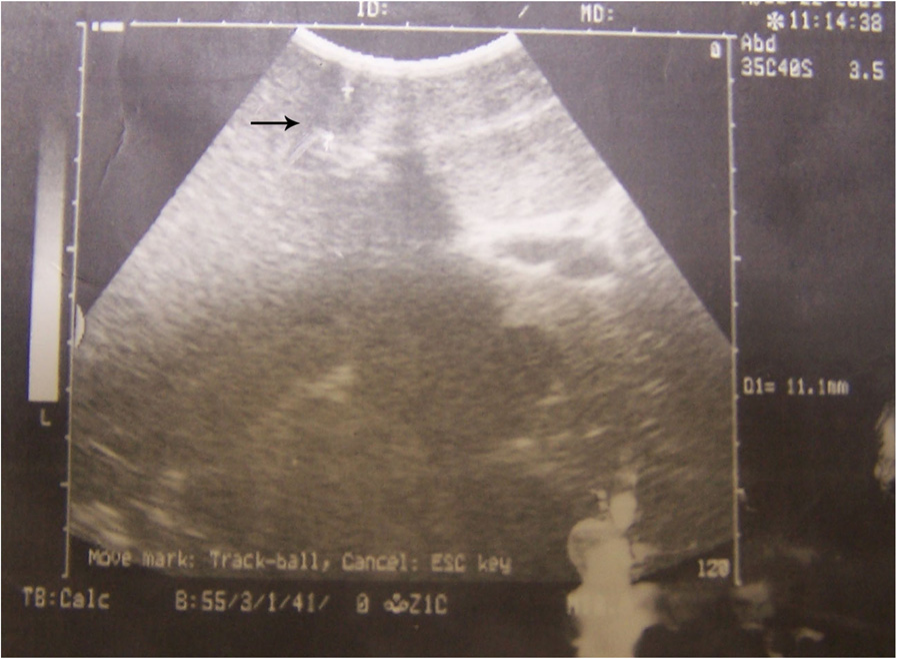
**Intraoperative findings.** Intraoperative ultrasonography. The arrow indicates the location of the pseudolesion.

**Figure 4:**
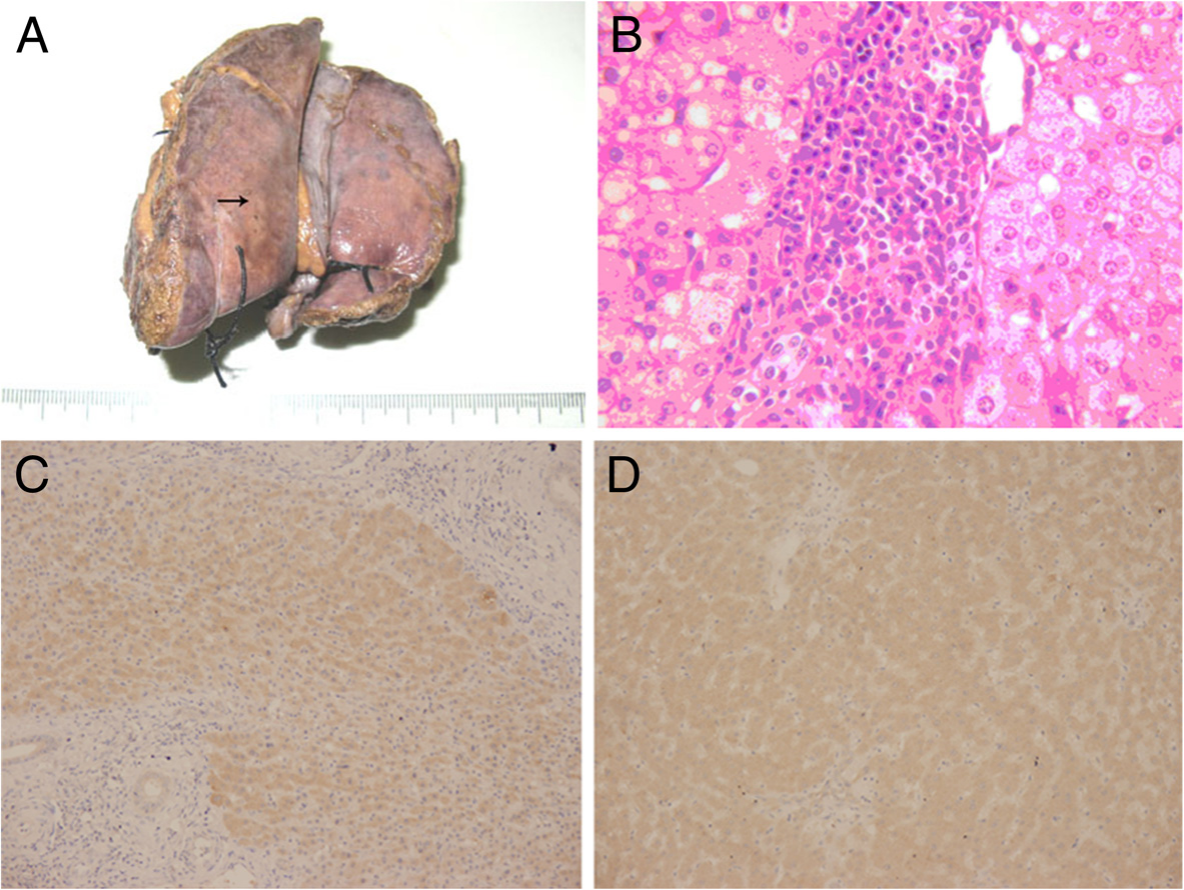
**Pathological findings. (A)** An image of the gross hepatic specimen. The arrow indicates the pseudolesion. **(B)** The pathology sample showed chronic hepatitis and liver regeneration (hematoxylin and eosin staining; magnification × 200). **(C)** Alpha fetoprotein (AFP) in nodular regenerative hyperplasia tissue (immunohistochemical staining; magnification × 200). **(D)** AFP in normal tissue (immunohistochemical staining; magnification × 200).

**Figure 5:**
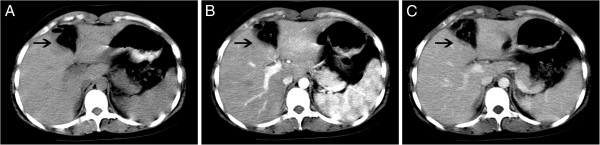
**Postoperative computed tomography (CT) findings.** In each panel, the arrow indicates the operative site. **(A)** Unenhanced phase CT image. **(B)** Arterial-dominant phase CT image. **(C)** Portal-dominant phase CT image.

We discussed the finding with the patient, who agreed to be admitted and undergo digital subtraction angiography (DSA) to rule out obvious vascular malformation or a malignant lesion. The lesion was not obviously enhanced in DSA, and no clear blood supply or arterial-portal fistula was found (Figure 2). After further discussion with the patient (and as she knew magnetic resonance imaging (MRI) might not safely tell the lesion with greatly elevated AFP from a malignant one), she declined to await MRI and demanded a biopsy of the liver and suspected lesion. To avoid possible metastasis and a false-negative result by needle biopsy, she agreed to laparoscopic hepatectomy without Pringle’s hepatic inflow occlusion and intraoperative ultrasonography (Figure 3). A wedge-shaped hepatic specimen, 3 × 5 × 5 cm, was removed and sent for pathological analysis (Figure 4A). Examination of the frozen section showed no malignant cells. The formal postoperative pathology results were as follows (Figure 4B): 1) chronic hepatitis; 2) nodular regenerative hyperplasia and focal nodular hyperplasia of the liver; 3) mild vascular malformation; and 4) HbsAg(−), HbcAg(−). Immunohistochemistry was performed, but no difference of AFP expression was found between hepatic lesion (Figure 4C) and normal tissue (Figure 4D). No surgical complications were seen. On postoperative day 7, the patient had Child A liver function and an AFP of 8,377 ng/ml. It was accepted that the elevated AFP resulted from primary chronic hepatitis instead of a malignant hepatic lesion. The patient was transferred to the Department of Internal Medicine with a final diagnosis of hepatic pseudolesion and primary non-viral (B, C, or G) chronic hepatitis. She received protocol including dexamethasone and Compound Glycyrrhizin in the Department of Internal Medicine. After another 2 weeks of steroid and liver protection therapy, the patient was discharged with an AFP of 46 ng/ml and Child A liver function. One month after hepatectomy,no hepatic lesion was found by CT in the postoperative follow-up (Figure 5).

## Discussion

Hepatic pseudolesion detected by CT that is complicated primarily by elevated AFP is confusing since it is difficult to rule out a malignant lesion using routine non-invasive procedures. However, excessively invasive procedures are associated with increased health risks. Ideally, clinical procedures should confirm the diagnosis while minimizing invasion and cost.

Abnormal AFP with normal liver function and negative viral evidence were the major factors that were confusing in this case. AFP is produced by normal gastrointestinal cells, yolk sac cells, and fetal hepatocytes after birth [5]. AFP levels above 20 ng/mL are considered indicative of possible liver disease [6]. Among liver cirrhosis cases without hepatocelluar carcinoma (HCC), AFP is reported to be elevated in 20% and to exceed 100 ng/mL in only 3% [7]. An elevated AFP level has high diagnostic significance for HCC, since the probability of HCC is >90% at an AFP level of >200 ng/mL. However, the AFP level can also be elevated due to other neoplastic or non-neoplastic conditions [8].

### Diseases outside the liver that are associated with elevated AFP

Elevated AFP is a typical sign of pregnancy and teratoma [9,10]. When hepatitis B virus, hepatitis C virus, and hepatitis G virus are negative and liver function is normal, diseases outside the liver associated with elevated AFP, especially malignancies leading to hepatic metastasis, should be excluded first [11]. Some laboratory tests, such as human chorionic gonadotropin, ultrasonography, and radiography, may help exclude misdiagnoses.

### Non-invasive and micro-invasive procedures for suspected hepatic pseudolesion

Ultrasonography is the first test that should be performed when a hepatic lesion is suspected. Some hepatic pseudolesions, such as the one caused by rib compression during CT, may be negative in non-invasive transabdominal ultrasound [12]. In the case presented here, the hepatic pseudolesion was missed by non-invasive ultrasound but finally found by intraoperative ultrasonography. Intraoperative ultrasonography is more sensitive than non-invasive ultrasound, but it is limited because it is more invasive [13]. A negative result from non-invasive ultrasonography did not result in a final diagnosis but did suggest the possibility of hepatic pseudolesion.

CT and CT arterial portography images show hepatic pseudolesions as areas of low attenuation with a triangular shape located adjacent to the falciform ligament; these findings may indicate a pseudolesion with focal fatty infiltration [14]. However, only 24.2% of pseudolesion images persist in all phases of CT, and it may be difficult to differentiate pseudolesions from other lesions [1]. In the case we present, the hepatic lesion resulted from hepatic focal nodular hyperplasia and mild vascular malformation, and its appearance on CT was as an area of low attenuation that was adjacent to the falciform ligament. This led us to suspect primary pseudolesion.

MRI can be used to detect a pseudolesion [15], and high-quality 3 T MRI has been reported to be helpful in diagnosing a hypervascularpseudolesion [16]. Out-of-phase T1 weighted MRI images show a loss of signal in the pseudolesion that is related to the presence of microscopic fat [17]. However, fat deposition and vascular malformation in MRI could not be safely distinguished from a malignant lesion [18]. Chronic hepatitis and abnormal AFP further complicated the case. Thus, in this case the patient rejected to await MRI. However, the final pathological result in this case showed nodular regenerative hyperplasia and focal nodular hyperplasia of the liver that might be found by MRI [19]. Thus, regardless of hepatitis and abnormal AFP, MRI is recommended to search for evidence of hepatic pseudolesion.

DSA is a micro-invasive procedure that is usually utilized to diagnose and treat HCC. Although a false negative for HCC by DSA is a possibility, DSA can differentiate a hepatic pseudolesion caused by vascular malformation from a malignant lesion [20]. Unfortunately, in the case presented here there was focal nodular hyperplasia and only mild vascular malformation; thus, none of the typical signs of vascular malformation were found by DSA.

Although AFP levels might be elevated in patients with chronic liver disease, it seemed difficult to differentiate a benign tumor with abnormal AFP from a malignant hepatic lesion using only laboratory tests and non-invasive or micro-invasive procedures. Here, hepatic biopsy had to be performed since pathological results were needed to make a precise diagnosis.

### Hepatic biopsy

In the case reported here, the lesion observed on the CT images was interpreted as a suspected malignant lesion after we performed laboratory tests, non-invasive transabdominal ultrasound, and DSA. Laparoscopic hepatectomy without the Pringle maneuver and hepatic inflow occlusion was performed to avoid a false negative and possible implantation metastasis due to needle-core biopsy [21]. The lesion was small, adjacent to the inferior surface of the liver, and located by intraoperative ultrasonography. Because of these factors, hepatectomy did not carry risks of severe surgical complications such as hemorrhage, biliary fistula, and liver dysfunction. However, the final pathological results revealed that the elevated AFP was caused by primary non-viral (B, C, or G) chronic hepatitis instead of the hepatic lesion. Downregulation of AFP resulted from steroid and liver protection therapy for hepatitis instead of surgery. Needle-core biopsy followed by dynamic follow-up surveillance may be less invasive than hepatectomy and more appropriate for this kind of hepatic lesion. Needle-core biopsy, including percutaneous liver biopsy and transjugular liver biopsy, is mostly used to obtain a pre-operative specimen that is useful for both HCC diagnosis and grading [22]. Percutaneous liver biopsy is appropriate to use for hepatic lesions that can be located by non-invasive ultrasonography, CT, or MRI [23]. For hepatic pseudolesion complicated by primary non-viral (B, C, or G) hepatitis and abnormal AFP, regardless of whether the hepatic lesion can be biopsied, liver biopsy without contraindications is recommended to further characterize the hepatic disease.

## Conclusion

Here we present a case of hepatic pseudolesion observed by CT that was complicated by primary non-viral chronic hepatitis (B, C, or G) with elevated AFP. This pseudolesion may have been caused by focal nodular hyperplasia of the liver plus mild vascular malformation. This condition can present as areas with low attenuation at all stages of enhanced CT. It is typically adjacent to the falciform ligament and negative on non-invasive ultrasonography and DSA.

Heightened awareness of hepatic pseudolesion complicated by primary elevated AFP will help physicians avoid unnecessary invasive procedures. Diseases outside the liver that present with elevated AFP should be excluded first. MRI is recommended to provide clues of pseudolesion. Hepatic biopsy and follow-up surveillance rather than hepatectomy are recommended when non-invasive and micro-invasive procedures cannot provide enough evidence for a final diagnosis. More knowledge on primary non-viral (B, C, or G) chronic hepatitis with greatly elevated AFP levels and Child A liver function is in need. Additional procedures that are less invasive are needed to better diagnose and treat hepatic pseudolesion complicated by primary hepatitis with abnormal AFP.

## Consent

Written informed consent was obtained from the patient for publication of this Case report and any accompanying images. A copy of the written consent is available for review by the Series Editor of this journal.

## Ethical considerations

The patient provided permission to publish this report, and the report was approved by the Ethics Board of the Third Affiliated Hospital of Southern Medical University, China.

## Abbreviations

AFP: Alpha fetoprotein; CT: Computed tomography; DSA: Digital subtraction angiography; HCC: Hepatocellular carcinoma; MRI: Magnetic resonance imaging.

## Competing interests

The authors declare that they have no competing interests.

## Authors’ contributions

XLL collected clinical data and composed the manuscipt. FQL reviewed the paper. Clinical treatment was performed under instruction from LXL and HYC. LYZ and YHL analyzed the images. QZW was responsible for the result of pathological consultation. All authors read and approved the final manuscript.
